# Encrypted Spiking Neural Networks Based on Adaptive Differential Privacy Mechanism

**DOI:** 10.3390/e27040333

**Published:** 2025-03-22

**Authors:** Xiwen Luo, Qiang Fu, Junxiu Liu, Yuling Luo, Sheng Qin, Xue Ouyang

**Affiliations:** 1 Guangxi Key Lab of Brain-Inspired Computing and Intelligent Chips, School of Electronic and Information Engineering, Guangxi Normal University, Guilin 541004, China; lxwrenai@gmail.com (X.L.); j.liu@ieee.org (J.L.); yuling0616@gxnu.edu.cn (Y.L.); qinsheng@gxnu.edu.cn (S.Q.); ouyangxue@gxnu.edu.cn (X.O.); 2 Key Laboratory of Nonlinear Circuits and Optical Communications, Guangxi Normal University, Education Department of Guangxi Zhuang Autonomous Region, Guilin 541004, China

**Keywords:** spiking neural network, differential privacy, correlation-based privacy budget

## Abstract

Spike neural networks (SNNs) perform excellently in various domains. However, SNNs based on differential privacy (DP) protocols introduce uniform noise to the gradient parameters, which may affect the trade-off between model efficiency and personal privacy. Therefore, the adaptive differential private SNN (ADPSNN) is proposed in this work. It dynamically adjusts the privacy budget based on the correlations between the output spikes and labels. In addition, the noise is added to the gradient parameters according to the privacy budget. The ADPSNN is tested on four datasets with different spiking neurons including leaky integrated-and-firing (LIF) and integrate-and-fire (IF) models. Experimental results show that the LIF neuron model provides superior utility on the MNIST (accuracy 99.56%) and Fashion-MNIST (accuracy 92.26%) datasets, while the IF neuron model performs well on the CIFAR10 (accuracy 90.67%) and CIFAR100 (accuracy 66.10%) datasets. Compared to existing methods, the accuracy of ADPSNN is improved by 0.09% to 3.1%. The ADPSNN has many potential applications, such as image classification, health care, and intelligent driving.

## 1. Introduction

In recent years, machine learning has gained significant attention in various fields, such as image classification [1,2], traffic signal control [3,4], and speech recognition [5]. Artificial neural network (ANN) is a crucial branch of machine learning, which has shown significant performance improvements [6,7]. Spiking neural networks (SNNs) [8,9] as the third generation of ANNs, operate in an event-driven manner. Information between layers is transmitted through binary spikes using biological neuron models. SNNs offer advantages such as low power consumption, high robustness, and fast inference speed. They also provide good biological interpretability and hold significant potential for application in neuromorphic hardware [10]. The training datasets for SNN may contain sensitive personal information such as secure passwords, genomic data, and more. However, due to the current limitations of privacy protection mechanisms, it is difficult for SNNs to achieve an optimal balance between performance and privacy. To resist attacks without compromising performance, the development of robust privacy algorithms is crucial.

Differential privacy (DP) [11,12] is an effective privacy protection mechanism. It provides personalized privacy protection against potential attackers, even for the scenarios where attackers have access to all other records except the target record. DP provides various specific implementation solutions tailored to different privacy protection requirements and analysis tasks. The approach of [13] explores learning prior knowledge from images generated by random processes and transferring this prior knowledge to differential data to improve the privacy-utility trade-off of DP-SGD. The method of [14] can ensure both strict differential privacy and balanced computational accuracy of the model.

The inference risk of SNN members was significantly increased by introducing an input dropout strategy, exhibiting privacy vulnerabilities comparable to those of ANN [15]. To address this issue, the DPSNN framework was proposed in [16], providing powerful privacy guarantees for SNN models while maintaining high accuracy. In [17], the construction of low-power SNNs using pre-trained ANN was explored, avoiding the exposure of sensitive information in datasets. Further, an Encrypted-SNN was proposed in [18], addressing the privacy challenge during the transition from ANN to SNN. In the study of SNN privacy protection mechanisms, the impact of quantization and alternative gradient selection on SNN privacy protection was revealed in [19]. By integrating SNN models, the reliability of decision-making was maintained and the uncertainty quantification function of SNN was enhanced in [20]. A novel federated learning framework combining SNN and DP was proposed in [21], which reduced inference attack accuracy to 43% while maintaining low power consumption. A new approach for data privacy protection was provided while maintaining the practicality of the data in [22]. Additionally, the flexibility of SNN in handling damaged inputs was demonstrated, utilizing noise-induced stochastic resonance and dynamic synapses in [23]. A neural morphological visual bullying detection method based on spatiotemporal spike signals was proposed to achieve a balance between privacy protection and bullying recognition in [24]. Privacy issues in SNN-based variational autoencoders (VAEs) were first explored in [25], providing a privacy-preserving solution for image generation and reconstruction tasks.

However, the current privacy-preserving neural network models based on DP still have certain limitations. One of the main challenges is the imbalance between privacy protection and model utility. Traditional DP methods utilize a fixed amount of noise during input processing, which can result in an inadequate trade-off between privacy and utility. When too much noise is added, it may decrease the accuracy and utility of the model. Conversely, insufficient noise may not provide sufficient privacy protection. Therefore, an adaptive differential private SNN (ADPSNN) method has been proposed, which balances model utility and training data privacy, improving the model’s accuracy. In addition, we analyzed the impact of leaky integrated and fire (LIF) [26], and integrated and fire (IF) [27] neurons on the applicability of ADPSNN by selecting the most suitable neuron model for different tasks and datasets. Overall, the three contributions of this work are summarized below.

- The proposed ADPSNN can adaptively adjust the amount of Gaussian noise added to the gradient. Specifically, it adds more noise to gradients that are weakly correlated with the model output to protect model privacy. Meanwhile, less noise is added to gradients that are strongly correlated with the model output to maintain model performance. This provides a more accurate method for protecting the training data privacy in SNNs;
- The performance impact of IF and LIF neurons on ADPSNN is analyzed. On the CIFAR10 and CIFAR100 datasets, the performances of IF neurons are 1.25% and 0.7% higher than that of LIF neurons, respectively. It aims to help select the most suitable neuron model for different tasks and datasets, thus enhancing the applicability of the approach;
- Our method has been extensively tested on four datasets to validate the feasibility of ADPSNN. It effectively balances privacy protection and the practicality of sensitive data in SNNs. In terms of model accuracy, our approach is improved by 0.09% to 3.1% compared to other existing methods. Therefore, our method may be used in some privacy-preserving scenarios, such as image classification, healthcare, and intelligent driving.

The remaining sections of the paper are organized as follows. A review literature on DP and SNN is provided in Section 2. The detailed methodology used in this work is described in Section 3. Experimental results and evaluation are given in Section 4, and Section 5 concludes the paper.

## 2. Preliminaries and Background

In this section, SNN along with the LIF and IF neuron models are introduced in Section 2.1. The DP, global sensitivity, and Gaussian noise mechanisms are introduced in Section 2.2.

### 2.1. Spiking Neural Network

**Figure 1:**
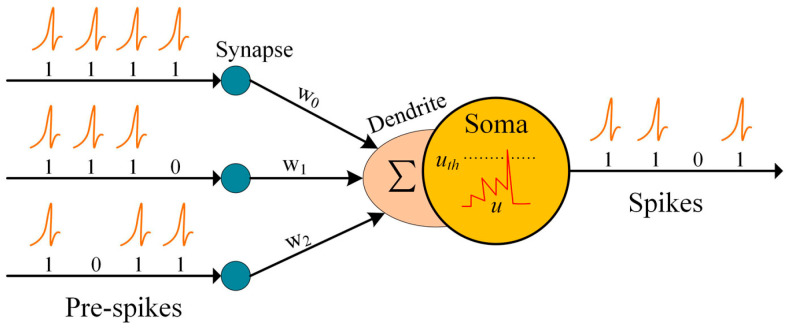
Computational model of SNN.

In a SNN, neurons play a fundamental role in transmitting information through discrete spiking events. It consists of three parts, i.e., dendrite, soma, and axon, as shown in Figure 1. Neurons are connected by synapses. When a neuron receives input from other neurons through its synapses, the input is weighted by the strength of each synapse. This weighted input is then transmitted to the dendrites of the neuron. The soma part of the neuron maintains and updates the membrane potential based on the magnitude of the weighted presynaptic input received from the dendrites. The membrane potential represents the electrical charge across the neuronal membrane and reflects the neuron’s state. As the soma accumulates and processes the incoming inputs, the membrane potential gradually changes. Once the membrane potential crosses a certain threshold set by the neuron, a spiking event, or spike, is generated within the neuron. This spike can be seen as a discrete electrical signal.

The LIF model is a widely used model for SNN, which simply describes the behavior of each biological neuron. The LIF model can be calculated by

(1) $$\left\{\begin{array}{l} \tau \frac{du(t)}{dt}=-u\left(t\right)+\sum_{j}w_{j}o_{j}(t)\\ o\left(t\right)+=Dirac\left(t-t^{\prime }\right)\\ u\left(t^{\prime }\right)=u_{0} \end{array}\right.$$

where t is the time step, τ is the time constant, u is the membrane potential, and o is the output spike. The output spike of the jth neuron is denoted by $o_{j}$, and $w_{j}$ stands for the synaptic weight between jth and the current neuron. The reset potential following the spike is denoted by $u_{0}$, while the trigger threshold is represented by $u_{th}$. In the continuous-time domain of the LIF model, the Dirac function can serve as a representation of the spiking signal. This function exhibits a peak of infinite height at the firing time, representing the firing spike event of the neuron. By modulating the membrane potential of a neuron through integrating spikes, one can interpret the neuron’s input as a form of current and integrate it into the neuron’s membrane potential. Upon surpassing a predetermined threshold, the neuron produces a spike and transmits it to other neurons connected to it. After that, the membrane potential is reset to its initial potential in preparation for receiving the next input.

The IF model is another classical method to describe the neuronal dynamics of SNN. The cell membrane is treated as a capacitor and the dynamics of the IF neuron can be governed by differential equations. A neuron fires and returns to its resting potential $u_{rest}$ when the membrane potential exceeds a threshold $u_{th}$. The IF model can be calculated by

(2) $$\left\{\begin{array}{l} \tau \frac{du(t)}{dt}=-[u\left(t\right)-u_{rest}]+\alpha \sum_{j}w_{j}o_{j}(t)\\ u\left(t^{\prime }\right)=u_{0} \end{array}\right.$$

where $u\left(t\right)$ is the dynamic change in each neuron’s membrane potential, $u_{rest}$ is the resting potential, $\sum_{j}w_{j}o_{j}(t)$ is the input received by each neuron, α is the response coefficient of each neuron to the input, and τ is the time constant of each neuron’s membrane potential.

The convolutional SNN can incorporate a network architecture that includes convolutional, pooling, and fully connected layers, akin to convolutional neural networks. However, the input to a convolutional SNN differs significantly; it can either be spike event data captured via dynamic vision sensors or spike inputs derived from Bernoulli sampling transformation applied to standard image datasets. Unlike traditional neural networks, which output continuous values via activation functions, convolutional SNN require classification based on the spike signals within the output layer. The timing and frequency of spikes serve as the basis for representing various pattern classes. This method of spike encoding allows convolutional SNNs to handle temporal information, offering clear benefits for event-driven tasks. By merging traditional convolutional, pooling, and fully connected layers within its architecture, the convolutional SNN provides a versatile and robust framework for utilizing spike signals in classification tasks, encoding and processing temporal data, and serving it as an efficacious tool for managing dynamic event-driven data. This study is designed to benchmark the performance of SNN against conventional approaches in the realm of static image recognition, a domain that has been extensively explored in prior research until the emergence of SNN [28].

### 2.2. Differential Privacy

DP aims to protect data confidentiality by introducing noise or altering the original dataset. This obfuscation hampers the ability of adversaries to ascertain the presence of specific entries. Even in scenarios where an attacker has comprehensive access to all but one particular record, DP ensures that the existence of that singular record within the database remains indiscernible. The definition of DP provides a well-calibrated trade-off between personal privacy and data utility, offering a measurable degree of privacy assurance. It mandates that any modification to the dataset should exert a negligible effect on the outcomes of analyses that are not specific to any one individual. Consequently, DP upholds the privacy of individuals while maintaining the integrity and precision of the overall dataset.

**Definition** **1** **(*ε*-Differential Privacy [11]).** *Given a pair of adjacent datasets D and $D^{\prime }$ ∈D, an algorithm that is random M: D→R meets the requirements for ε-DP if and only if for any subset of outputs O⊆R, the probability $Pr\left(M\left(D\right)\in O\right)$ can be calculated by*

(3) $$Pr\left(M\left(D\right)\in O\right)\leq e^{\varepsilon }Pr\left(M\left(D^{\prime }\right)\in O\right)$$

*where Pr represents probability. This condition ensures that the probability ratio for the occurrence of a particular output subset O when executing an algorithm M on different datasets D and $D^{\prime }$. The probability ratio does not exceed $e^{\varepsilon }$. Privacy budget ε is a crucial factor influencing the strength of privacy protection. It represents the level of privacy protection offered by the algorithm M. A smaller privacy budget ensures stronger privacy guarantees, implying that the algorithm M produces consistent results and does not significantly change when any record is removed from the database. The privacy budget is typically chosen to be small. It is crucial to recognize that a non-zero privacy budget is necessary. In theory, a zero privacy budget would provide the highest level of protection for sensitive data during training. In this scenario, the model would produce uniformly probable indistinguishable outcomes for all adjacent dataset pairs, stripping the output of any meaningful insights about the data. However, a zero privacy budget undermines the balance between model utility and the protection of data privacy. ε-differential privacy offers a precise definition of privacy protection. It is noteworthy to emphasize, nonetheless, that (ε,δ)-differential privacy is a more lenient variant of ε-differential privacy. The parameter δ, which is invariably set to a value greater than zero (δ > 0), quantifies the probability of a privacy breach even under stringent DP conditions. (ε,δ)-differential privacy denotes the high chance of ε-differential privacy being attained by a randomized method, which is contingent upon the value of δ. The definition of (ε,δ)-differential privacy is provided by*

(4) $$Ρr\left(M\left(D\right)\in O\right)\leq e^{\varepsilon }Ρr\left(M\left(D^{\prime }\right)\in O\right)+\delta$$

*where ε-differential privacy is excessively stringent. This privacy analysis concept is built on the Gaussian Differential Privacy (GDP) framework proposed by Dong et al. [29]. In GDP, a mechanism M is deemed to satisfy μ-GDP if the distribution of its output on the dataset D cannot be discerned from the distribution of Gaussian noise N(μ,1), which has a mean of μ and a standard deviation of one, in any statistically significant manner.*

Gaussian noise is widely used in DP. It is used to input noise to the DP mechanism to preserve the privacy of sensitive data. The value range of Gaussian noise is in the field of real numbers, which can generate continuous random numbers and better match the characteristics of actual data. By adjusting the standard deviation of Gaussian noise through the global sensitivity and privacy budget, the size of the noise can be flexibly controlled, and the balance between privacy protection and data utility can be achieved. The Gaussian noise introduced by this mechanism is independently generated on each occasion, uninfluenced by prior noise or data inputs, thereby guaranteeing the mechanism’s reliability and uniformity. Global sensitivity stands as a critical metric in determining the requisite noise level for DP. It is the maximum variation of a function f over all possible datasets D. Within the Gaussian mechanism, this global sensitivity is instrumental in determining the scale of noise to be added, facilitating a delicate balance between the privacy of the data and its utility. As a rule of thumb, an increase in global sensitivity necessitates the integration of a greater quantum of noise.

**Definition** **2** **(Global sensitivity [11]).** *The global sensitivity is calculated depending on the specific function $f:D\to R^{d}$. For example, for a query function f, global sensitivity can be obtained by computing the maximum absolute difference of the function f over different datasets $D^{\prime }$, which is defined as*

(5) $$GS_{f}\left(D\right)=\underset{D,D^{\prime }}{\max}{\left||f\left(D\right)-f(D^{\prime })|\right|}_{1},$$

*where D and $D^{\prime }$ are any two neighbor databases, and ${\left||f\left(D\right)-f(D^{\prime })|\right|}_{1}$ is the $l_{1}$ distance between $f\left(D\right)$ and $f(D^{\prime })$. By determining the global sensitivity, combined with privacy parameters such as ε, an appropriate standard deviation of Gaussian noise can be chosen to achieve the desired level of privacy protection.*

**Definition** **3** **(Gaussian mechanism [11,29]).** *The Gaussian mechanism is a common privacy protection method, that introduces random noise to the data to protect the privacy of sensitive information. For any δϵ(0,1), there is $Y∼N(0,\sigma^{2})$ makes mechanism M that satisfies (ε,δ)-DP. It can be calculated by*

(6) $$M\left(D\right)=f\left(D\right)+Y,$$

*where δ is the standard deviation of the Gaussian noise mechanism. δ determines the amount of noise inputted into the data. However, the privacy budget ε has a negative correlation with the noise level, that is, a lower privacy budget means a larger noise scale is inputted. The relaxation, denoted by the symbol σ, refers to the risk probability that a violation of strict requirements for data privacy protection occurs. It can be calculated by*

(7) $$\sigma >\frac{\sqrt{2ln(1.25/\delta )GS_{f}\left(D\right)}}{\varepsilon }.$$

## 3. ADPSNN

A novel privacy-preserving paradigm for SNN based on correlation analysis is given in this section. The promoted method safeguards the privacy of the training dataset while maintaining the model’s effectiveness. This is achieved by the strategic infusion of noise into the gradient. To be more precise, the gradient is modified by the proposed mechanism, which employs the Gaussian mechanism in conjunction with a correlation analysis method, adjusting it adaptively in response to the relationship between the output spike and the corresponding label.

### 3.1. The ResNetSNN Architecture

**Figure 2:**
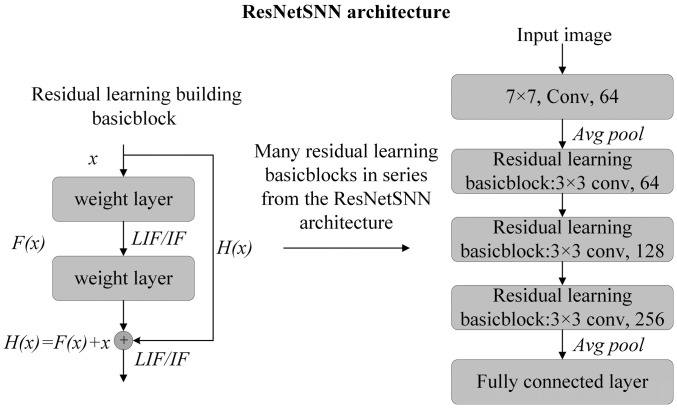
The architecture of the ResNetSNN model.

In the research of [30], a ResNet model with LIF neuron is selected for the classification task. Our work combines ResNet and LIF with IF spiking neurons to construct a ResNetSNN model. The detailed structure of the ResNetSNN model is depicted in Figure 2. The ResNetSNN network architecture is underpinned by a fundamental building block known as the residual block, which is employed by neural networks to facilitate residual connections. This structure can ameliorate the model’s performance by mitigating the issues of gradient explosion and vanishing in SNNs. Initially, the input data is processed through a convolutional layer for feature extraction, then it is normalized by a batch normalization layer, which accelerates the training process and bolsters the model’s generalization capabilities. Following this, the data undergo an activation function such as LIF/IF, augmenting the model’s non-linear representation. Finally, the implementation of the residual connection is completed by adding the output of a skip connection to the processed data. In this case, the input data is represented by x, the residual function by F(x), the original output by H(x), and the realization of the residual connection by F(x)+x. Instead of learning the original output H(x) directly, the neural network can learn the residual function F(x) with this design. This enhances the model’s performance by enabling the neural network to utilize the input data’s information more effectively. This work uses a ResNetSNN18 architecture for the MNIST [31] and Fashion-MNIST [32] datasets, while the CIFAR10 and CIFAR100 datasets use a ResNetSNN19 architecture.

### 3.2. Gradient Perturbation for SNN

In this research, a distinctive ADPSNN mechanism is devised to ensure privacy for SNN. This mechanism modulates the quantity of Gaussian noise infused into the gradient, thereby not only successfully protecting the privacy of the data in the training set, but also preserving the operational efficacy of the model.

**Figure 3:**
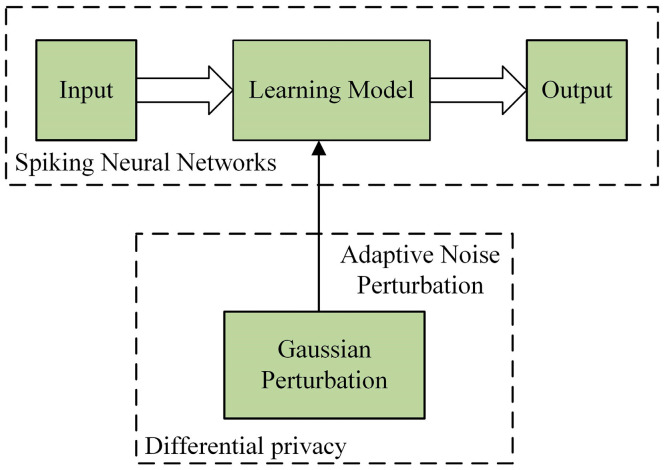
Diagram of the overall ADPSNN mechanism.

The ADPSNN mechanism combines the advantages of SNN and DP as shown in Figure 3, which is able to improve the performance of SNN while protecting individual privacy. The mechanism consists of three main components: input, learning model, and output. In the input phase, the data are pre-processed and entered into the learning model, which is constructed by the ResNetSNN. The structure of a neural network consists of multiple neurons, each receiving input from other neurons and computing its output, which is modeled using SNN-based models. In this mechanism, the gradient parameter is passed by inputting noise adaptively. This approach allows neurons to inject noise while passing gradient parameters, thereby preserving privacy. DP is realized by Gaussian privacy. Gaussian privacy is a DP algorithm that protects data privacy by inputting Gaussian noise, which is inputted adaptively to balance the needs of privacy protection and neural network performance.

A set *L* of randomly selected training samples from the dataset *D* is used in each training step. Updating the parameter ω for the mini-batch dataset in t steps, starting from the initial point $\omega_{0}$ by

(8) $$\omega_{t+1}=\omega_{t}-\eta_{t}\left(\lambda \omega_{t}+\frac{1}{L}\sum_{x_{i}\in L_{t}}\nabla L\left(\omega_{t},x_{i}\right)\right),$$

where λ is the regularization parameter, $\eta_{t}$ is the learning rate at step t, and $\nabla L\left(\omega_{t},x_{i}\right)$ is the gradient over the sample.

To provide a learning mechanism for SNN that meets the requirements of ε-differential privacy, noise is incorporated into the gradient update procedure. This mechanism updates the parameter ω at the DP step t by

(9) $$\omega_{t+1}=\omega_{t}-\eta_{t}\left(\lambda \omega_{t}+\frac{1}{L}\left(\sum_{x_{i}\in L_{t}}g\left(x_{i}\right)+n_{t}\right)\right),$$

where the Gaussian noise vector, $n_{t}$, is random.

Introducing a fixed amount of noise into the gradient can influence the learning outcomes of the model, potentially disrupting the equilibrium between the model’s effectiveness and the privacy protection of the training data. This research suggests the ADPSNN mechanism as a solution to enhance the model’s performance. Depending on the correlation between the model output spike and the label, this ADPSNN can introduce noise in an adaptable manner. First, the correlation $C_{i}(D)$ of the model output spikes and labels can be computed for the i-th data, which can be calculated by

(10) $$C_{i}(D)=\frac{\sum_{i=1}^{n}\left(X_{i}-\bar{X})(Y_{i}-\bar{Y}\right)}{\sqrt{\sum_{i=1}^{n}(X_{i}-\bar{X}{)}^{2}}\sqrt{\sum_{i=1}^{n}(Y_{i}-\bar{Y}{)}^{2}}},$$

where $X_{i}$ represents the prediction result of the neural network for the i-th data, and $X_{i}\in D$, $Y_{i}$ represents the true label for the i-th data, and n represents the total number of data in dataset D. $\bar{X}$ and $\bar{Y}$ are the average prediction overall data and the average actual label overall data, respectively.

To ensure $C_{i}(D)\in [0,1]$, each $C_{i}(D)$ is normalized to $Nor\_C_{i}(D)$ by

(11) $$Nor\_C_{i}(D)=\frac{C_{i}(D)-\beta }{\eta -\beta },$$

where η and β denote the maximum and minimum value, respectively, in $\left\{C_{1}\left(D\right),C_{2}\left(D\right),\ldots ,C_{d}\left(D\right)\right\}$. Next, an adaptive Gaussian noise strategy is used by a relevance ratio $\alpha_{i}$. This means that gradients with a lower correlation to the model output receive more noise, while gradients with a higher correlation receive less noise. It can be calculated by

(12) $$\alpha_{i}=\frac{C_{i}}{\sum_{i=1}^{d}C_{i}},$$

and the privacy budget $\varepsilon_{i}$ is calculated by

(13) $$\varepsilon_{i}=\left(1-\alpha_{i}\right)\times \varepsilon ,$$

Last, Gaussian noise $N(0,\sigma^{2})$ is inputted to the gradient for protection.

**Theorem** **1.** *Suppose the DP can run via Equation (9) for T batches $L_{1},L_{2},...,L_{T}$, where the batches $L_{t}$ are disjoint. If ${\left\|g\right.\left.\left(x_{i}\right)\right\|}_{1}\leq 1$ for all ω and $\left(x_{i},y_{i}\right)$, ε-differential privacy is satisfied by using its additional Gaussian noise.*

**Proof.**  Let L and $L^{\prime }$ be two neighboring batches. Let the parameters on L and $L^{\prime }$ be denoted by $\omega_{t+1(L)}$ and $\omega_{t+1(L^{\prime })}$, which can be calculated by

(14) $$\omega_{t+1(L)}=\omega_{t}-\eta_{t}\left(\lambda \omega_{t}+\frac{1}{L}\sum_{x_{i}\in L_{t}}g(x_{i})\right),$$

(15) $$\omega_{t+1(L^{\prime })}=\omega_{t}-\eta_{t}\left(\lambda \omega_{t}+\frac{1}{L}\sum_{{x^{\prime }}_{i}\in {L^{\prime }}_{t}}g({x^{\prime }}_{i})\right),$$

and the inequality can be calculated by

(16) $$\Delta_{\omega_{t}}=\frac{\eta_{t}}{\left|L\right|}\sum_{\omega \in \omega_{t}}{∥\sum_{x_{i}\in L_{t}}g\left(x_{i}\right)-\sum_{x_{i}^{\prime }\in L_{t}^{\prime }}g\left(x_{i}^{\prime }\right)∥}_{1}=\frac{\eta_{t}}{\left|L\right|}\sum_{\omega \in \omega_{t}}({∥g\left(x_{1}\right)∥}_{1}-\;{∥g\left(x_{1}^{\prime }\right)∥}_{1})\leq \frac{\eta_{t}}{\left|L\right|}\sum_{\omega \in \omega_{t}}\left({∥g\left(x_{1}\right)∥}_{1}+{∥g\left(x_{1}^{\prime }\right)∥}_{1}\right)\leq \frac{\eta_{t}}{\left|L\right|}\sum_{\omega \in \omega_{t}}(\underset{x_{i}\in L_{t}}{\max}{∥g\left(x_{i}\right)∥}_{1}+\;\underset{x_{i}^{\prime }\in L_{t}^{\prime }}{\max}{∥g\left(x_{i}^{\prime }\right)∥}_{1})\leq \frac{\eta_{t}}{\left|L\right|}\sum_{\omega \in \omega_{t}}\left(2\cdot \underset{x_{i}\in L_{t}}{\max}{∥g\left(x_{i}\right)∥}_{1}\right)\leq 2\frac{\eta_{t}}{\left|L\right|}\underset{x_{i}\in L_{t}}{max}\sum_{\omega \in \omega_{t}}{∥g\left(x_{i}\right)∥}_{1},$$

where $\forall x_{i}:{\left\|g\right.\left.\left(x_{i}\right)\right\|}_{1}\leq 1$, $\sum_{x_{i}\in L_{t}}g\left(x_{i}\right)=g\left(x_{1}\right)+g\left(x_{2}\right)+\ldots +g\left(x_{n}\right)$, $\sum_{x_{i}^{\prime }\in L_{t}^{\prime }}g\left(x_{i}\right)=g\left(x_{1}^{\prime }\right)+g\left(x_{2}\right)+\ldots +g\left(x_{n}\right)$. Because L and $L^{\prime }$ are two neighboring batches, only one piece of data is different, therefore $\sum_{x_{i}\in L_{t}}g\left(x_{i}\right)-\sum_{x_{i}^{\prime }\in L_{t}^{\prime }}g\left(x_{i}^{\prime }\right)=g\left(x_{1}\right)-g\left(x_{1}^{\prime }\right)$. In addition, $\left\|g\left(x_{1}\right)\right\|\leq \underset{x_{i}\in L_{t}}{\max}{∥g\left(x_{i}\right)∥}_{1}$, and $\left\|g\left(x_{1}^{\prime }\right)\right\|\leq \underset{x_{i}\in L_{t}}{\max}{∥g\left(x_{i}^{\prime }\right)∥}_{1}$. This leads to the inequality ${∆}_{w_{t}}\leq 2\frac{\eta_{t}}{\left|L\right|}$. □

To achieve DP protection, the gradient can be inputted to adaptive noise while computing $\omega_{t+1}$, which is calculated by

(17) $$\omega_{t+1}=\omega_{t}-\eta_{t}\left(\lambda \omega_{t}+\frac{1}{L}\left(\sum_{x_{i}\in L_{t}}g\left(x_{i}\right)+N(0,\sigma^{2})\right)\right),$$

where the perturbation of the gradient is $\sum_{x_{i}\in L_{t}}g\left(x_{i}\right)+N(0,\sigma^{2})$. Then the inequality can be calculated by

(18) $$\begin{array}{l} \frac{Pr\left\{\omega_{t+1(L)}\right\}}{Pr\left\{\omega_{t+1\left(L^{\prime }\right)}\right\}}\\ =\frac{\prod_{\omega \in \omega_{t}}\prod_{j=1}^{d}exp\left(\frac{\varepsilon_{j}\frac{\eta_{t}}{\left|L\right|}{∥\sum_{x_{i}\in L_{t}}g\left(x_{i}\right)-\left(\sum_{x_{i}\in L_{t}}g\left(x_{i}\right)+N(0,\sigma^{2})\right)∥}_{1}}{\Delta_{\omega_{t}}}\right)}{\prod_{\omega \in \omega_{t}}\prod_{j=1}^{d}exp\left(\frac{\varepsilon_{j}\frac{\eta_{t}}{\left|L\right|}{∥\sum_{x^{\prime }\in L^{\prime }t}g\left(x_{i}^{\prime }\right)-\left(\sum_{x_{i}\in L_{t}}g\left(x_{i}\right)+N(0,\sigma^{2})\right)∥}_{1}}{\Delta_{\omega_{t}}}\right)}\\ \leq \prod_{\omega \in \omega_{t}}\prod_{j=1}^{d}exp\left(\frac{\varepsilon_{j}\frac{\eta_{t}}{\left|L\right|}}{\Delta_{\omega_{t}}}{∥\sum_{x_{i}\in L_{t}}g\left(x_{i}\right)-\sum_{x^{\prime }\in L^{\prime }t}g\left(x_{i}^{\prime }\right)∥}_{1}\right)\\ \leq \prod_{\omega \in \omega_{t}}\prod_{j=1}^{d}exp\left(\frac{\varepsilon_{j}\frac{\eta_{t}}{\left|L\right|}}{\Delta_{\omega_{t}}}2{max}_{x_{i}\in L_{t}}{∥g\left(x_{i}\right)∥}_{1}\right)\\ \leq exp\left(\sum_{\omega \in \omega_{t}}1\cdot \sum_{j=1}^{d}\varepsilon \frac{2\eta_{t}\alpha_{i}}{|L|\Delta_{\omega_{t}}}\right)\\ \leq exp\left(\varepsilon \frac{2\eta_{t}\left[\sum_{j=1}^{d}\alpha_{i}\right]}{{|L|\Delta }_{\omega_{t}}}\right)\\ =exp\left(\varepsilon \right), \end{array}$$

where $a_{i}={max}_{x_{i}\in L_{t}}{\left\|g\left(x_{i}\right)\right\|}_{1}=\frac{C_{i}}{\sum_{i=1}^{d}C_{i}}$, $\Delta_{\omega_{t}}=2\frac{\eta_{t}}{|L|}\underset{x_{i}\in L_{t}}{max}\sum_{\omega \in \omega_{t}}{\left\|g\left(x_{i}\right)\right\|}_{1}=2\frac{\eta_{t}}{|L|}{\left[\sum_{j=1}^{d}\alpha_{i}\right]}_{i}$.

## 4. Experiments and Results

### 4.1. Datasets

The CIFAR100, CIFAR10, MNIST, and Fashion-MNIST datasets are widely used for image classification tasks. The CIFAR100 contains 100 different categories. Each category includes a wide variety of objects, animals, plants, vehicles, and scenes, making it a diverse dataset. There are 600 training and 100 test images in each category, each measuring 32 by 32 pixels. The CIFAR10 is a small dataset containing 60,000 color images in total. A training set of 50,000 photos and a test set of 10,000 images are the two subgroups into which it is divided. The MNIST dataset comprises approximately 70,000 grayscale images, each sized 28 × 28 pixels; 60,000 of them are used to train the model and another 10,000 to test the performance of the model. The images include handwritten digits from 0 to 9 with approximately 7000 sample images per digit. The Fashion-MNIST dataset is a dataset containing fashion-related clothing and accessories images for testing and validating image classification algorithms. It has the same number of categories, total amount of data and sample dimensions as the MNIST dataset. In addition, the summary of hardware, software, and hyperparameters information was completed to ensure reproducibility in Table 1.

**Figure 4:**
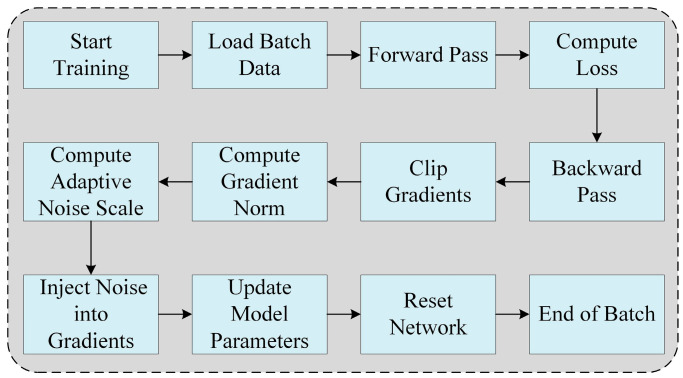
Adaptive noise injection gradient flowchart during training process.

In the training process, adaptive noise is injected into the gradients as shown in Figure 4. After computing the gradients during the backward pass, the gradient norm is calculated. Based on the correlation between the model’s output and the labels, as well as the differential privacy parameters, an adaptive noise scale is dynamically determined. This noise scale is then used to generate Gaussian noise, which is added to the gradients. The noisy gradients are applied to update the model parameters, ensuring both differential privacy and improved robustness. Finally, the network is reset to prepare for the next batch or timestep.

### 4.2. Experiments on Differentially Private Algorithms

**Figure 5:**
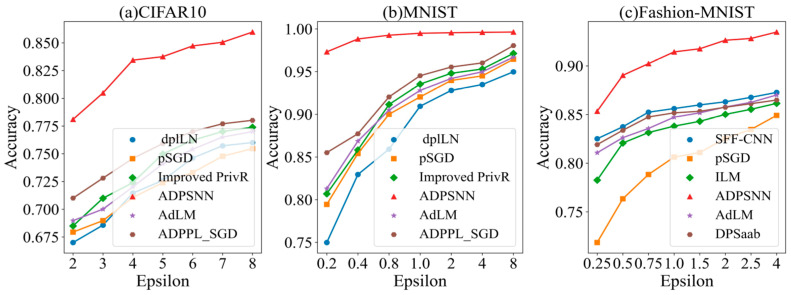
The test accuracy of the ADPSNN in comparison to competing models.

Figure 5 illustrates that the ADPSNN exhibits higher accuracy than other algorithms under different privacy budgets on the CIFAR10, MNIST, and Fashion-MNIST datasets. On the CIFAR10 dataset, the ADPSNN surpasses the cutting-edge ADPPL_SGD [33] optimization method by a margin of 7.95% in accuracy, attaining a peak accuracy of 85.97%, which is more advantageous than the DP SGD algorithm with Identical Laplace Noise (dpILN), pSGD [34], Improved PrivR [35], and AdLM [36] methods. On the MNIST dataset, the accuracy is higher than that of other algorithms. When the privacy budget is set to two, the ADPSNN’s accuracy advantage over the ADPPL_SGD reaches its zenith at 11.80%, and this margin of superiority incrementally widens as the privacy budget is expanded. With the privacy budget set to eight, the mechanism’s test accuracy soars to 99.63%, outstripping the ADPPL_SGD by 1.59% and setting a new benchmark for state-of-the-art performance. On the Fashion-MNIST dataset, the performance is better than the SFF-CNN [37], pSGD, ILM [36], AdLM, and DPSaab [37] methods. As the privacy budget is incrementally enhanced, a corresponding gradual improvement in accuracy is observed. When the privacy budget is two, the approach tested in this study outstrips the prevailing superior SFF-CNN algorithm [33] by a substantial margin of 6.33%, achieving an impressive peak accuracy of 92.64%. Figure 5 shows that the ADPSNN maintains its utility and high accuracy, outperforming alternative algorithms when operating under identical privacy-preserving constraints.

### 4.3. Experiments on Different Privacy Levels

**Figure 6:**
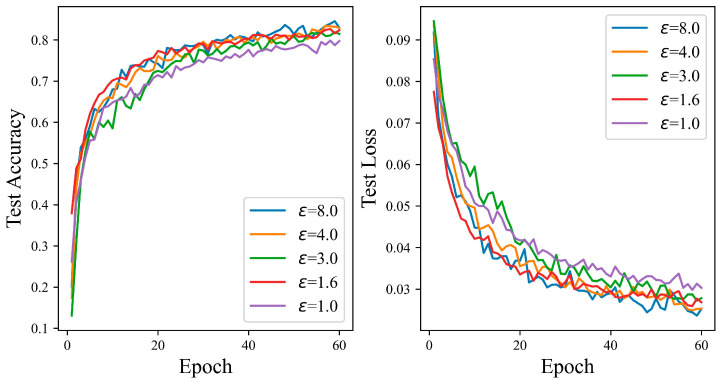
The test accuracy of the ADPSNN for different epsilons on the CIFAR10 dataset.

Figure 6 delineates a positive correlation between the accuracy of the proposed mechanism increases and the increment of the privacy budget on the CIFAR10 dataset. When the privacy budget is one, the test accuracy is generally lower than other values. As the number of iterations increases, there is a notable uptick in accuracy across the spectrum of privacy budgets. After conducting 60 iterations at privacy budgets of 1.6, 3.0, and 4.0, the test accuracies are 77.58%, 82.21%, and 82.40%, respectively. When the privacy budget is eight, the ADPSNN attains its pinnacle of accuracy at 83.95%, which is a significant improvement of 4.08% over the accuracy observed at the lowest budget of one.

**Figure 7:**
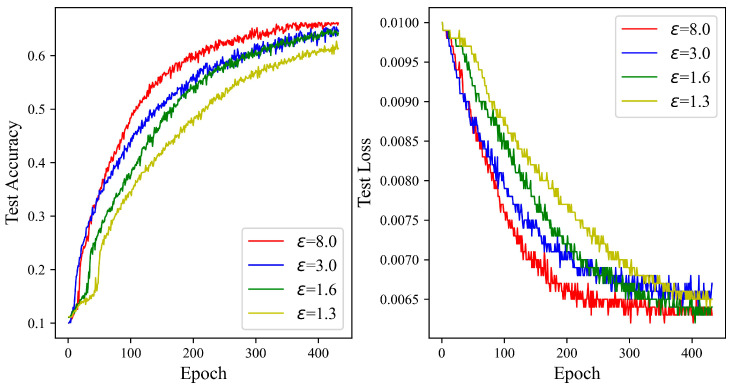
The test accuracy of the ADPSNN for different epsilons on the CIFAR100 dataset.

Figure 7 illustrates a general upward trend in the test accuracy curve in tandem with increases in the privacy budget. A gradual climb in accuracy is witnessed when the privacy budget is set to 1.3, cresting at 62.67%. The zenith of accuracy is recorded at 65.42% with a privacy budget of 8.0, marking a 2.75% enhancement over the budget of 1.3. At privacy budgets of 1.6 and 3.0, the accuracies attained are 64.92% and 65.22%, respectively. The experimental results underscore the ADPSNN mechanism’s capacity to maintain robust performance on the complex CIFAR100 dataset while affording a substantial degree of data privacy protection.

**Figure 8:**
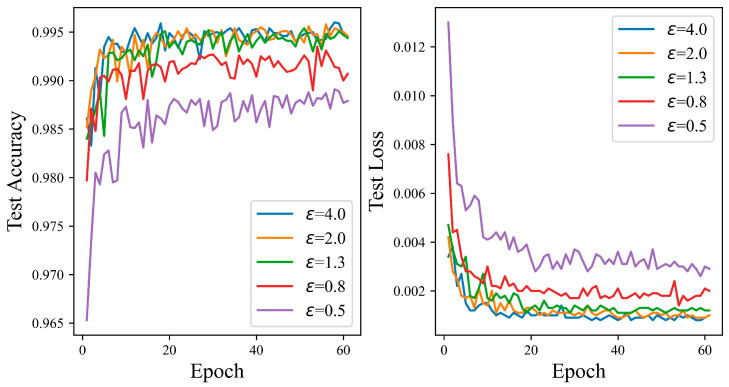
The test accuracy of the ADPSNN for different epsilons on the MNIST dataset.

Figure 8 demonstrates that as the budget for privacy rises, the test accuracy curve of the proposed mechanism generally rises. When the privacy budget is 0.5, the SNN has the lowest test accuracy. However, it still achieves a high accuracy of 98.91%, indicating that even if more gradient perturbations are inputted into the SNN network, it can maintain good performance. The suggested algorithm performs at 0.44% as the privacy budget rises to 0.8, higher than that achieved with a privacy budget of 0.5, and remains relatively stable. Finally, as the privacy budget reaches four, the test accuracy peaks at 99.60%. Overall, Figure 8 shows that the ADPSNN mechanism can maintain relatively high performance on the MNIST data set while protecting sensitive information.

**Figure 9:**
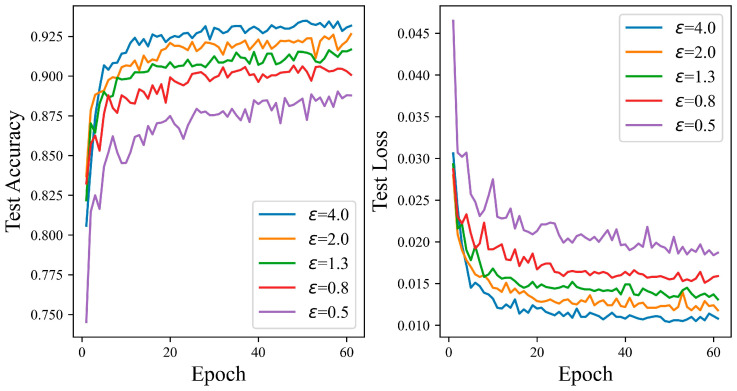
The test accuracy of the ADPSNN for different epsilons on the Fashion-MNIST dataset.

Figure 9 shows that the test accuracy curves of the proposed mechanism all rise in tandem with the increase in the privacy budget, and this rising trend is obvious. When the privacy budget is 0.5, the test accuracy curve of SNN is the lowest as a whole, but it still achieves a high accuracy of 89.03%. When the privacy budget is 0.8, 1.3, 2.0, and 4.0, the accuracy reaches 90.66%, 91.67%, 92.64%, and 93.48%, respectively. When the privacy budget is four, the performance is 4.45% higher than that of 0.5. With the increase in the number of iterations, the test accuracy curve remains relatively stable. The findings of the experiment confirm the high effectiveness of ADPSNN, and the ADPSNN can maintain good performance while protecting sensitive information.

### 4.4. Experiments on Different SNN Algorithms

Table 2 shows that the ADPSNN is better than other SNNs. On the MNIST dataset, the test accuracy of the LIF model reaches 99.56%, which is higher than that of other methods: 0.93% higher than DPSNN [16], 0.09% higher than the Encrypted-SNN method in [18], 0.56% higher than the method of [38], and 0.34% higher than the approach of [19]. The training accuracy of the IF model reaches 99.47%, which is slightly lower (0.09%) than the LIF model, and the time step is eight in the training process. The ADPSNN can protect against sensitive information leakage while maintaining good performance. On the MNIST dataset, the LIF model performs better than the IF model.

Table 3 indicates that the accuracy of the IF neuron model is 89.42%, the accuracy of the LIF neuron model tested is 90.67%, and the time step is eight on the CIFAR10 dataset. Compared with other training algorithms without privacy protection, the performance is close to 90.00%. However, on the IF and LIF neuron models, the accuracy is 1.37% and 0.12% higher than the PrivateSNN training method in [17], and 2.57% and 1.32% higher than the Encrypted-SNN method [18], respectively. Compared with methods [15,19], the proposed method improved the performance of LIF neurons by 10.43% and 1.22%, respectively. Table 3 shows that the ADPSNN can strike a balance between the model’s usefulness and performance on the CIFAR10 dataset, and the IF performs better than the LIF neuron model.

**Figure 10:**
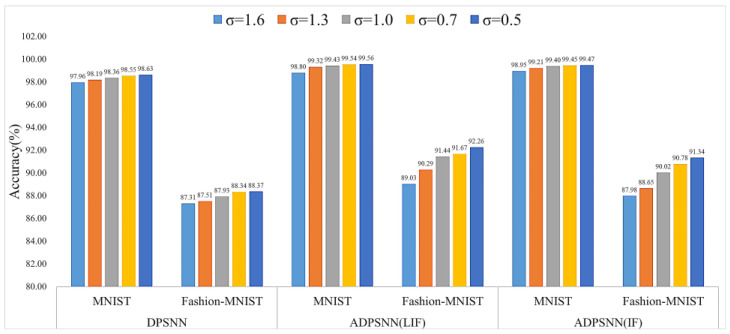
Performance comparisons of different studies on MNIST and Fashion-MNIST datasets with different noise scales.

Figure 10 illustrates that the performance of the ADPSNN is better than the DPSNN privacy protection method on the LIF and IF neuron models. Whether it is on the MNIST dataset or the Fashion-MNIST dataset, the performance bar charts increase with the reduction in the inputted noise scale. The degree of increase on the MNIST dataset is slightly slower than the degree of increase on the Fashion-MNIST dataset. The MNIST dataset is much simpler and less complex than the Fashion-MNIST dataset. When the noise scale is 1.6, the accuracy of the proposed ADPSNN mechanism on the MNIST dataset is 98.80% using the IF neuron model. When using the IF neuron model, the accuracy is 98.95%, which is higher than the accuracy of the DPSNN method of 97.96%. Figure 10 reveals that, across noise scales of 1.3, 1.0, 0.7, and 0.5, the LIF model consistently outperforms the IF model in test performance on both the MNIST and Fashion-MNIST datasets within the ADPSNN framework. This suggests that the LIF model is more effective than the IF neuron model in processing these datasets.

Table 4 shows the effectiveness of the proposed ADPSNN mechanism. This study is based on the training methods described in the references [10,17,18,46]. Results indicate that the performance of ADPSNN is stable in the CIFAR100 dataset. The ADPSNN using IF and LIF neuron models achieved 66.1% and 65.4% on the CIFAR100 dataset, respectively. ADPSNN (LIF) and ADPSNN (IF) are 2.4% and 3.1% higher than the Encrypted-SNN [18] method on the CIFAR100 dataset, 3.1% and 3.8% higher than the PrivateSNN [17] method, and 1.5% and 2.2% higher than the method of [15], respectively. This means that ADPSNN can maintain high performance while protecting sensitive information on the CIFAR100 dataset.

### 4.5. Experiments on the LIF and IF Models

**Figure 11:**
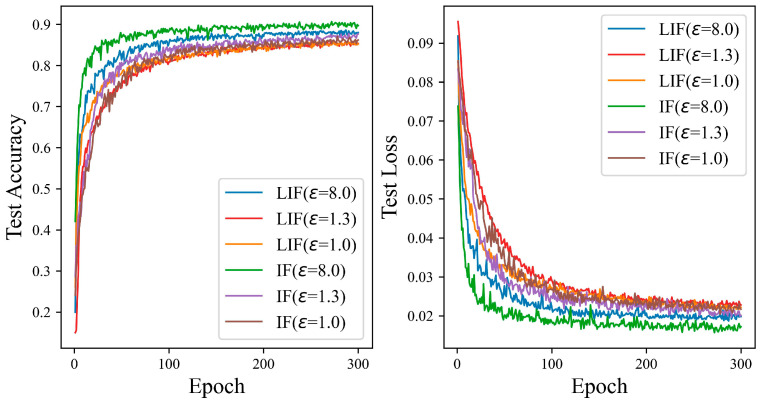
The test accuracy of the ADPSNN using LIF and IF neurons for different epsilons on the CIFAR10 dataset.

Figure 11 shows that the ADPSNN uses the IF neuron model to train and test the data on the CIFAR10 dataset. The performance is better than that of the LIF neuron model. When the privacy budget is one, the LIF neuron model initially exhibits superior performance, with its test accuracy surpassing that of the IF neuron model as the number of iterations increases. However, this trend reverses around the 95th iteration, where the IF neuron model’s test accuracy curve begins to exceed that of the LIF neuron. At higher privacy budgets of 1.3 and 8.0, the test accuracy of the IF neuron model is markedly superior to that of the LIF neuron model, with the discrepancy in test accuracy reaching 1.99% and 2.04%, respectively. These experimental results validate the feasibility of the mechanism proposed in this paper. Particularly for the intricate CIFAR10 dataset, the IF neuron model demonstrates enhanced performance compared to the LIF neuron model. The findings affirm that both neuron models can uphold high test accuracy while providing robust privacy safeguards.

**Figure 12:**
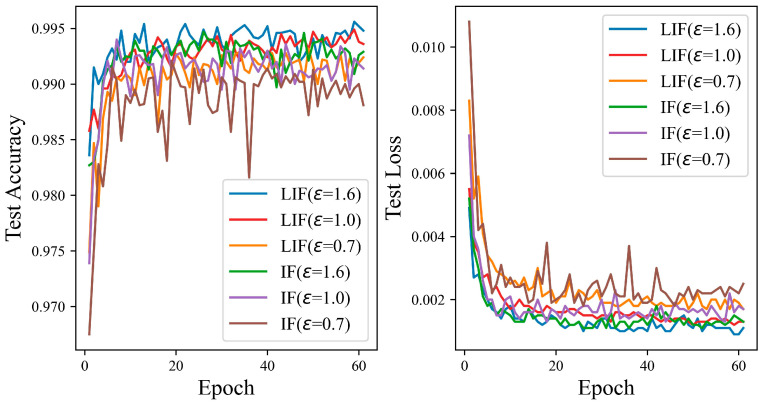
The test accuracy of the ADPSNN using LIF and IF neurons for different epsilons on the MNIST dataset.

Figure 12 shows that the test accuracy of the LIF neuron model is better than that of the IF neuron model on the MNIST dataset. The test curve of the IF neuron is relatively smoother, and the fluctuation is not as large as that of the LIF neuron model. When the privacy budgets are set to 0.7 and 1.0, the test curve and loss curve of the IF model fluctuate greatly, due to additional Gaussian noise, which affects the model’s performance in some way. At privacy budget settings of 0.7, 1.0, and 1.6, with the iteration count held at 60, the disparity in test accuracy between the IF and LIF models is observed to be 0.43%, 0.22%, and 0.19%, respectively. The test accuracy of the LIF neuron model can reach 99.56%.

**Figure 13:**
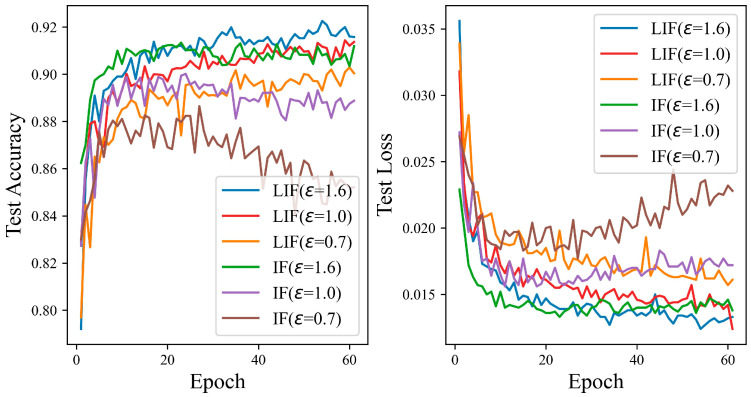
The test accuracy of the ADPSNN using LIF and IF neurons for different epsilons on the Fashion-MNIST dataset.

Figure 13 indicates that the LIF neuron model’s performance is better than that of the IF neuron model on the Fashion-MNIST dataset. As the privacy budget is elevated, the test accuracy curve for the LIF neuron model generally exhibits an upward trajectory. In contrast, for the IF neuron model, the performance curve demonstrates a decline as the number of iterations increases at privacy budgets of 0.7 and 1.0, with the descent being more pronounced with the 0.7 privacy budget than with 1.0. Specifically, at privacy budgets of 0.7, 1.0, and 1.6, the test accuracy achieved by the ADPSNN mechanism on the LIF model reaches 90.27%, 91.44%, and 92.26%, respectively. Meanwhile, the IF neuron model under the same mechanism achieves test accuracies of 88.65%, 90.02%, and 91.34%. These experimental outcomes suggest that the ADPSNN mechanism better harmonizes model utility with privacy protection, particularly when applied to the Fashion-MNIST dataset.

## 5. Conclusions

To solve the issue of SNN models balancing data privacy protection and high performance, this work proposes an adaptive DP protection learning mechanism for SNN models, namely ADPSNN. ADPSNN not only dynamically introduces Gaussian noise to meet the requirements of DP, but also dynamically adjusts the noise intensity added to the gradient based on correlation. The strength of ADPSNN lies in dynamically analyzing the correlation between the spike sequence output by the model and the real labels, thereby achieving precise control over noise allocation. For gradients that are weakly correlated with the model output, ADPSNN tends to apply higher levels of noise to effectively blur sensitive information and enhance data privacy protection. On the contrary, the amount of noise should be moderately reduced to avoid unnecessary damage to model performance. In addition, this work examines the performance of LIF and IF neural models under the ADPSNN mechanism and analyzes the performance and DP effect of neural models in processing complex classification tasks. Experimental results demonstrate that ADPSNN can maintain high model performance while ensuring data privacy, achieving a balance between the two and providing strong support for the application of SNN in privacy-sensitive scenarios.

In the future, the advancement of the ADPSNN mechanism will center on three key areas. Firstly, there will be an emphasis on refining the gradient adaptation strategy and implementing advanced algorithms to enhance learning convergence while deepening our comprehension of SNN mechanisms. Secondly, innovative fine noise injection techniques are being developed, effectively balancing privacy with performance. Lastly, our applications are planned to be expanded to key areas such as healthcare, finance, and intelligent transportation. They contribute to the innovation and development of SNN and differential privacy-preserving techniques by combining expertise in signal processing and cryptography. For example, in the healthcare field, ADPSNN can be applied for secure medical image analysis and patient data processing. In the financial field, it can enhance fraud detection systems while protecting transaction privacy. In intelligent transportation, it can realize real-time, privacy protected traffic monitoring and auto drive system.

## Figures and Tables

**Table 1:** Hardware, software, and hyperparameters information.

Category	Parameter	Value/Description
Hardware	GPU	NVIDIA RTX 3090 (24GB VRAM)
	CPU	13th Gen Intel(R) Core(TM) i5-13600K 3.50 GHz
	RAM	32 GB
	Storage	SSD
Software	Python	3.9
	Torch	2.3.1
	SpikingJelly	0.0.0.0.14
	Torchvision	0.18.0
	TensorBoard	2.18.0
	Optimizer	Adam
	Threshold	10
	Reset Potential	0.0
	Time Constant	2
	Surrogate Function	ATan
	Delta	1.0 × 10^−5^

**Table 2:** Performance of SNN on MNIST dataset.

Approaches	Neuron Model	Input Coding	Learning Rule	Accuracy (%)	Timesteps
2 FC [39]	LIF	Rate	STDP	95.00	700
784FC-600FC-10FC [40]	IF	Temporal	Backprop	96.98	167
36C3-2P-128FC-10FC [41]	LIF	Rate	Stochastic STDP	66.23	100
15C5-P2-40C5-P2-300FC [42]	LIF	Encoding layer	Backprop	99.53	5
LeNet-5 [43]	IF	Temporal	ANN-to-SNN	98.53	-
[16]	LIF	Temporal	DPSNN	98.63	-
[44]	LIF	-	Backprop	99.52	-
[38]	LIF	-	Backprop	98.91	300
ConvNet [19]	LIF	Temporal	Backprop	99.22 (±0.02)	25
VGG16 [18]	-	-	ANN-to-SNN	99.47	
ADPSNN (This work)	LIF	Poisson	Backprop	99.56	8
ADPSNN (This work)	IF	Poisson	Backprop	99.47	8

**Table 3:** Performance comparisons of different models on CIFAR10 datasets.

Approaches	Neuron Model	Input Coding	Learning Rule	Accuracy (%)	Timesteps
VGG16 [45]	IF	Temporal	ANN-to-SNN	93.63	2048
VGG16 [46]	IF	Rate	ANN-to-SNN	91.55	1000
VGG16 [47]	LIF	Rate	Hybrid	92.02	200
VGG9 [48]	IF	Rate	Backprop	90.45	100
256C3-2P-1024 FC-10FC [40]	LIF	Rate	Stochastic STDP	98.54	100
CIFARNet [49]	-	Encoding layer	Backprop	90.53	12
CIFARNet [50]	-	Encoding layer	Backprop	91.41	5
VGG16 [51]	-	Encoding layer	Hybrid	92.70	5
PrivateSNN [17]	LIF	-	ANN-to-SNN	89.30	-
ConvNet [19]	LIF	Temporal	Backprop	78.99 (±0.33)	25
ResNet18 [15]	LIF	Latency	ANN-to-SNN	88.20	4
ResNet18 [10]	-	-	ANN-to-SNN	94.91	16
VGG16 [52]	-	-	ANN-to-SNN	95.14	6
ResNet18 [52]	-	-	ANN-to-SNN	94.57	6
Encrypted-SNN [18]	-	-	ANN-to-SNN	88.10	-
ADPSNN (This work)	LIF	Poisson	Backprop	89.42	8
ADPSNN (This work)	IF	Poisson	Backprop	90.67	8

**Table 4:** Performance comparisons of different models on CIFAR100 datasets.

Approaches	Neuron Model	Input Coding	Learning Rule	Accuracy (%)	Timesteps
VGG16 [46]	IF	Rate	ANN-to-SNN	62.7	1000
PrivateSNN [17]	LIF	-	ANN-to-SNN	62.3	-
Encrypted-SNN [18]	-	-	ANN-to-SNN	63.0	-
ResNet18 [10]	-	-	ANN-to-SNN	68.1	16
ResNet18 [15]	LIF	Latency	ANN-to-SNN	63.9	4
ADPSNN(LIF) (This work)	LIF	Poisson	Backprop	65.4	8
ADPSNN(IF) (This work)	IF	Poisson	Backprop	66.1	8

## Data Availability

The data used to support the findings of this study are included within the article and are cited at relevant places within the text as references.
